# Low-Pressure Plasma-Processed Ruthenium/Nickel Foam Electrocatalysts for Hydrogen Evolution Reaction

**DOI:** 10.3390/ma15072603

**Published:** 2022-04-01

**Authors:** Chen Liu, Chia-Yun Tseng, Ying-Chyi Wang, I-Chun Cheng, Jian-Zhang Chen

**Affiliations:** 1 Graduate Institute of Applied Mechanics, National Taiwan University, Taipei City 10617, Taiwan; r09543009@ntu.edu.tw (C.L.); r10543033@ntu.edu.tw (C.-Y.T.); r10543002@ntu.edu.tw (Y.-C.W.); 2 Advanced Research Center for Green Materials Science and Technology, National Taiwan University, Taipei City 10617, Taiwan; 3 Department of Electrical Engineering, Graduate Institute of Photonics and Optoelectronics, National Taiwan University, Taipei City 10617, Taiwan; iccheng@ntu.edu.tw; 4 Innovative Photonics Advanced Research Center (i-PARC), National Taiwan University, Taipei City 10617, Taiwan; 5 Graduate School of Advanced Technology, National Taiwan University, Taipei City 10617, Taiwan

**Keywords:** hydrogen evolution reaction, plasma, catalyst, ruthenium (Ru), electrolysis

## Abstract

In this paper, low-pressure 95%Ar–5%H_2_, pure Ar, and 95%Ar–5%O_2_ plasmas were used for post-treatment of ruthenium (Ru) deposited on nickel foam (NF) (Ru/NF). Ru/NF was then tested as a catalyst for a hydrogen evolution reaction. Significant improvement in electrocatalytic activity with the lowest overpotential and Tafel slope was observed in an alkaline electrolyte (1 M KOH) with 95%Ar–5%O_2_ plasma processing on Ru/NF. Linear scanning electrical impedance spectroscopy (EIS) and cyclic voltammetry (CV) also indicate the lowest interfacial impedance and largest electrical double layer capacitance. Experimental results with 0.1 M phosphate buffered saline (PBS) and 0.5 M H_2_SO_4_ electrolytes were also demonstrated and compared.

## 1. Introduction

The global energy demand is continuously increasing owing to rapid industrial development. The global average annual power generation is approximately 2520 GW and annual growth rate of power generation is 2.5% [1]. The extensive use of fossil fuels, such as coal, oil, and natural gas, has dramatically increased carbon dioxide emissions and, in turn, made global warming an increasingly serious concern [2]. To mitigate global warming, alternative energy sources such as renewable energy are being actively explored owing to their smaller carbon footprint. Hydrogen energy is considered one of the most promising alternative energy sources. It involves the storage of energy in the form of chemical bonds and the use of fuel cells (or other equipment) to generate electricity for end-users when needed [3].

Water electrolysis with renewable energy is a green and economical method for producing hydrogen. Platinum (Pt) is the most commonly used catalyst for the hydrogen evolution reaction (HER) of water electrolysis. One main drawback of Pt is its high cost. Over the last 10 years, ruthenium (Ru) has, on average, been 13 times cheaper than Pt [4,5]. Further, Ru and Pt have chemical bonds of similar strength and Ru has a similar ability to dissociate water [6,7]. Therefore, in this study, we investigate Ru as a highly efficient and low-cost catalyst for HER. Previous studies have noted that oxygen (O_2_) plasma treatment can produce defects on the catalyst surface and create an oxygen-incorporated structure on the catalysts [8,9,10]. These improve the electrocatalytic activity of the catalysts. Some studies have shown that argon (Ar) or hydrogen (H_2_) plasma treatment has a similar effect [10,11]. In this study, we investigated an Ru catalyst on a nickel foam (NF) electrode (hereafter called the Ru/NF electrocatalyst) with different types of plasma treatments.

An ideal electrocatalyst for HER should work efficiently under a wide range of electrolyte pH values, just like traditional Pt-based catalysts [12,13,14]. Although neutral aqueous electrolytes are ecofriendly [15] and cause less damage to electrocatalysts, they have hardly been investigated [16]. Electrocatalysts in acidic aqueous electrolytes exhibit high efficiency for generating H_2_ and have therefore been widely investigated in recent years [17,18,19,20,21,22,23,24,25,26]. However, electrocatalysts in alkaline aqueous electrolytes are used most widely in the industry because the lower vapor pressure of the electrolyte solution makes them more economical [25,27]. Additionally, owing to the low-corrosion environment, the electrocatalysts have a longer lifetime [28]. In this study, low-pressure 95%Ar–5%H_2_, pure Ar, and 95%Ar–5%O_2_ plasmas were used for post-treatment of Ru/NF electrocatalysts that were then used for water electrolysis. Different electrolyte solutions with various pH values were tested.

## 2. Experimental Section

### 2.1. Materials and Regents

For this study, 1.7 mm-thick NF was purchased from HOMYTECH, Taiwan. Sulfuric acid (H_2_SO_4_, purity: 95−97%) and acetone (ACE, purity: 99%) were purchased from AUECC, Taipei City, Taiwan. Ethanol (purity: 95%) was purchased from Echo Chemical, Miaoli County, Taiwan. Ethylene glycol (EG, purity: 99%) was purchased from SHOWA, Tokyo, Japan. Ruthenium (III) chloride hydrate (RuCl_3_ H_2_O), potassium hydroxide (KOH, purity: 85%), and phosphate-buffered saline (PBS) powders were purchased from Sigma-Aldrich, St. Louis, MO, USA. Unless otherwise stated, all chemicals were used as received without any further purification.

### 2.2. Synthesis of Ru Electrocatalysts

The untreated NF is named pristine NF. NF (4.0 cm × 3.0 cm × 0.17 cm) was first immersed in a 0.1 M H_2_SO_4_ solution with ultrasonication for 20 min to remove native oxides on the surface. Then, the NF was sequentially immersed in de-ionized (DI) water, alcohol, and ACE with ultrasonication for 20 min [29]. The resulting NF is denoted as NF*. Then, 0.08 g of RuCl_3_ was dissolved in a solution consisting of 20 mL of EG and 20 mL of DI water. Subsequently, NF* was immersed in the solution inside a Teflon-lined autoclave that was sealed and heated at 90 °C for 2 h. The Ru on NF* was then rinsed with DI water and calcined in a furnace at 60 °C for 10 min [5]. The resulting electrode is denoted as Ru/NF*. Finally, low-pressure plasmas of different gases—pure Ar, 95% Ar + 5% H_2_, and 95% Ar + 5% O_2_—were used to post-treat Ru/NF*. The resulting products were named Ru/NF*—A, Ru/NF*—AH, and Ru/NF*—AO, respectively.

### 2.3. Characterization

X-ray diffraction (Bruker D2 PHASER XRD) was performed in the 2θ range of 10°–90° with Cu Kα radiation (λ = 0.154060 nm). The water contact angles were measured using a goniometer (Sindatek, Taipei City, Taiwan, Model 100SB). The morphology and chemical composition were characterized by scanning electron microscopy (SEM, JSM-IT100, JEOL) with energy-dispersive X-ray spectrometry (EDS) and X-ray photoelectron spectroscopy (XPS, Thermo Scientific, Waltham, MA, USA, Theta Probe). A low-pressure plasma machine (Harrick, Plasma Cleaner PDC-32G, New York, NY, USA) was used with a pressure of 0.6 torr, flow rate of 8 sccm, and power of 7 W. An electrochemical workstation (Autolab PGSTAT204, Metrohm, Utrecht, The Netherlands) was used to perform cyclic voltammetry (CV; −0.25–−0.05 V, potential scan speed: 20−300 mV/s), linear sweep voltammetry (LSV; scanning rate: 5 mV/s), and electrochemical impedance spectroscopy (EIS; 0.1–100,000 Hz) measurements in a three-electrode configuration to characterize the electrocatalyst. Ag/AgCl was used as the reference electrode and platinum (Pt) as the counter electrode. The potential E was then converted to the reverse hydrogen electrode (RHE) by E (vs. RHE) = E (vs. Ag/AgCl) + 0.059 × pH + 0.197 [30,31]. The HER performance of the electrocatalyst was evaluated in three different pH electrolytes. Then, 1 M KOH aqueous solution (pH ≈ 14), 0.5 M H_2_SO_4_ aqueous solution (pH ≈ 0), and 0.1 M phosphate buffer solution (PBS, pH ≈ 7) were used as the alkaline, acidic, and neutral electrolyte, respectively.

## 3. Results and Discussion

### 3.1. Water Contact Angle

**Figure 1:**
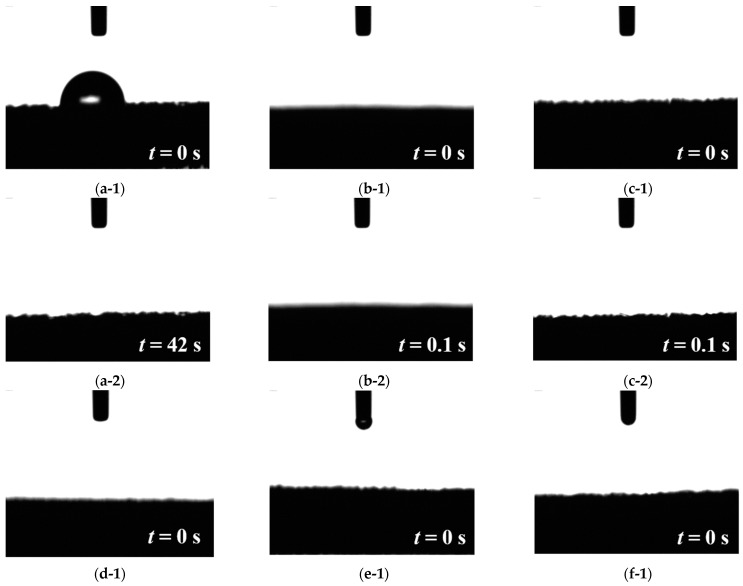

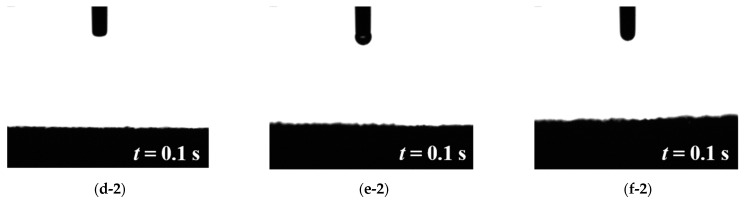
Water contact angles of (**a-1**) pristine NF right after the droplet was dispensed, (**a-2**) pristine NF after the droplet was dispensed for 42 s, (**b-1**) NF* right after the droplet was dispensed, (**b-2**) NF* after the droplet was dispensed for 0.1 s, (**c-1**) Ru/NF* right after the droplet was dispensed, (**c-2**) Ru/NF* after the droplet was dispensed for 0.1 s, (**d-1**) Ru/NF*-A right after the droplet was dispensed, (**d-2**) NF*-A after the droplet was dispensed for 0.1 s, (**e-1**) Ru/NF*-AH right after the droplet was dispensed, and (**e-2**) Ru/NF*-HO after the droplet was dispensed for 0.1 s; (**f-1**) Ru/NF*-AO right after the droplet was dispensed, and (**f-2**) Ru/NF*-AO after the droplet was dispensed for 0.1 s.

**Figure 2:**
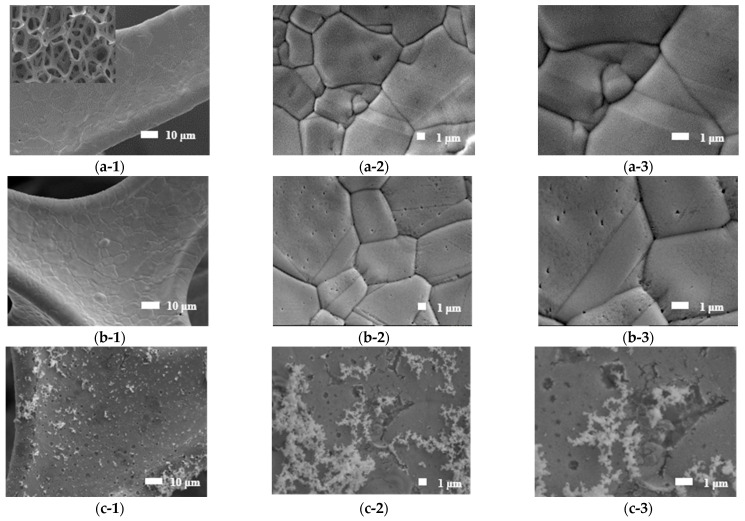

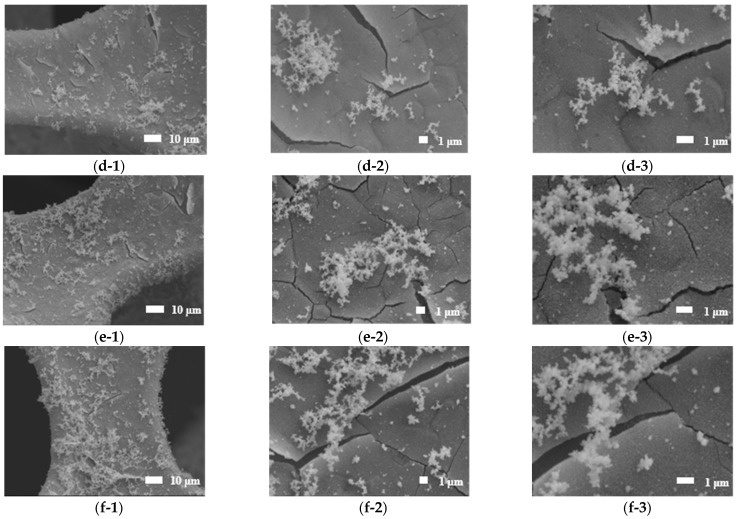
SEM images of pristine NF with (**a-1**) 1000×, (**a-2**) 5000×, and (**a-3**) 10,000× magnification; NF* with (**b-1**) 1000×, (**b-2**) 5000×, and (**b-3**) 10,000× magnification; Ru/NF* with (**c-1**) 1000×, (**c-2**) 5000×, and (**c-3**) 10,000× magnification; Ru/NF*-A with (**d-1**) 1000×, (**d-2**) 5000×, and (**d-3**) 10,000× magnification; Ru/NF*-AH with (**e-1**) 1000×, (**e-2**) 5000×, and (**e-3**) 10,000× magnification; and Ru/NF*-AO with (**f-1**) 1000×, (**f-2**) 5000×, and (**f-3**) 10,000× magnification.

Figure 1(a-1) shows the water contact angle of the pristine NF right after the droplet was dispensed. The water contact angle of pristine NF was 72.1°; after 42 s, the droplet completely penetrated the NF substrate, as shown in Figure 1(a-2). Figure 1b–f show the water contact angles of NF*, NF*, Ru/NF*, Ru/NF*-A, Ru/NF*-AH, and Ru/NF*-AO; the droplets immediately penetrated the H_2_SO_4_-treated NF and all plasma-treated Ru/NF*. This is attributed to the removal of the native oxide by H_2_SO_4_ treatment. This could reduce the electron transfer impedance. Further, better hydrophilicity could promote interfacial contacts between the electrolyte and the electrocatalysts [32], thus increasing the electrolyte–electrocatalyst interfacial reactive area and leading to improved performance.

### 3.2. SEM

Figure 2 shows the SEM images of the pristine NF, NF*, Ru/NF*, Ru/NF*-A, Ru/NF*-AH, and Ru/NF*-AO, respectively. The higher-magnification SEM images with pristine NF and NF* reveal the smooth structure of the surface Figure 2a,b. Figure 2c shows a thick Ru layer with a little fractal structure [5]. In comparison to Ru/NF*, the SEM images of the electrocatalysts with plasma treatment show a few more physical defects, especially more cracks, on the surface (Figure 2d–f).

### 3.3. XRD

**Figure 3:**
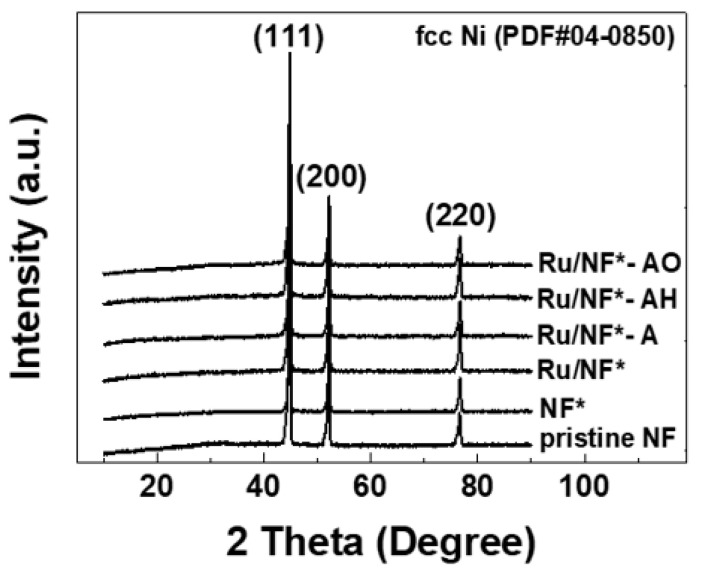
XRD patterns of the pristine NF, NF*, Ru/NF*, Ru/NF*-A, Ru/NF*-AH, and Ru/NF*-AO.

Figure 3 shows the XRD patterns of the pristine NF, NF*, Ru/NF*, Ru/NF*-A, Ru/NF*-AH, and Ru/NF*-AO. Strong diffraction peaks are seen at 44.5°, 52.0°, and 76.4°; these correspond to the face-centered cubic (FCC) structure of the NF [5,33]. No peaks of metallic ruthenium or ruthenium oxide were detected, probably because of their low content [5,34].

### 3.4. EDS and XPS

**Figure 4:**
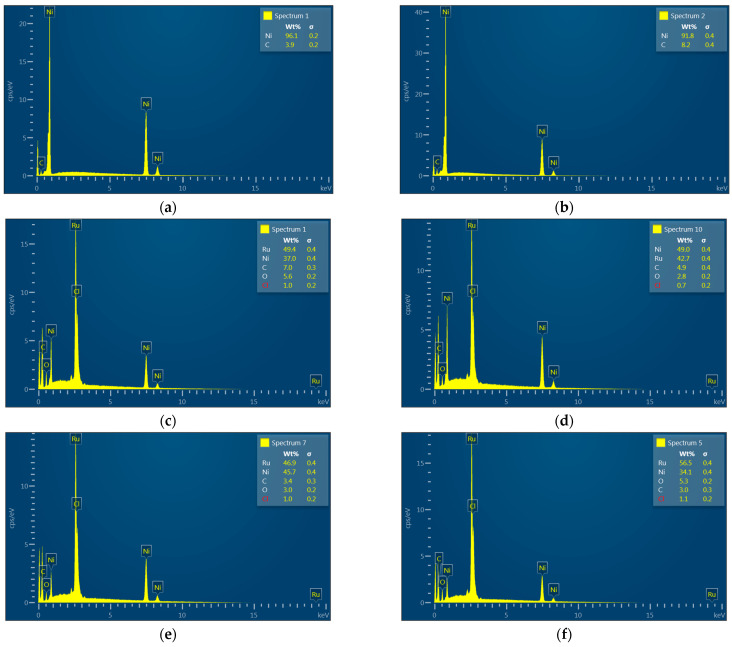
EDS spectra of (**a**) pristine NF, (**b**) NF*, (**c**) Ru/NF*, (**d**) Ru/NF*-A, (**e**) Ru/NF*-AH, and (**f**) Ru/NF*-AO.

Figure 4 and Table 1 show the EDS results. EDS results of Ru/NF*, Ru/NF*-A, Ru/NF*-AH, and Ru/NF*-AO reveal the deposition of an Ru layer on NF. The oxygen content in Ru/NF* decreased after 100% Ar and 95% Ar–5% H_2_ plasma treatment. Chemical elements were also determined by XPS. Figures S1–S4 (Supplementary Materials) show the XPS results. The Ru3d spectra and EDS spectra indicate the successful deposition of Ru. Because metallic nickel is more chemically active than Ru, the NF substrate can spontaneously act as an electron donor for the Ru layer. The accumulation of negative charges on the Ru surface can lead to a higher work function and increase the reduction activity, thereby accelerating the HER process [4,5]. Ru deposited on NF could greatly improve the HER, as discussed below in Section 3.5.

### 3.5. Evaluation of Electrocatalytic Activity

#### 3.5.1. KOH Electrolyte

**Figure 5:**
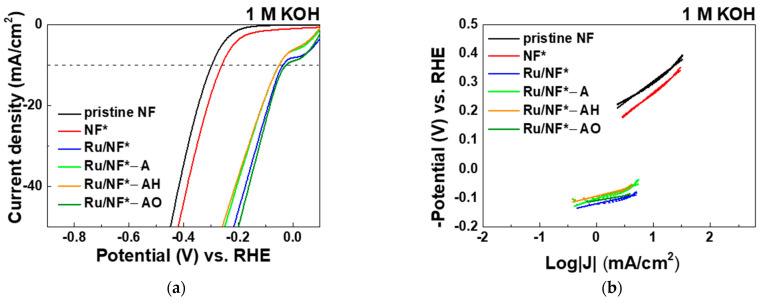

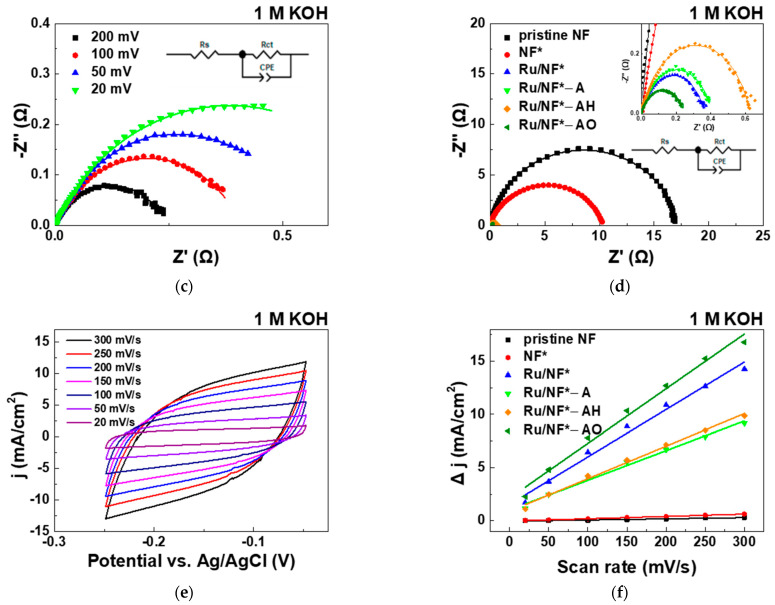
In 1 M KOH aqueous solution: (**a**) LSV polarization curves of pristine NF, NF*, Ru/NF*, Ru/NF*-A, Ru/NF*-AH, and Ru/NF*-AO catalysts toward HER. (**b**) Corresponding Tafel plots of HER. (**c**) Nyquist plots of Ru/NF*-AO catalyst at four different applied overpotentials versus RHE. (**d**) Nyquist plots of pristine NF, NF*, Ru/NF*, Ru/NF*-A, Ru/NF*- AH, and Ru/NF*-AO catalysts at an overpotential of 200 mV versus RHE. (**e**) CV potential curves of Ru/NF*-AO at different scan rates. (**f**) Current density of various electrocatalysts as a function of scan rate.

Figure 5 and Table 2 show the results of the electrochemical HER activity evaluated in 1 M KOH aqueous solution. Principally, the HER mechanism in a highly alkaline medium is described by the following three equations:

M + H_2_O + e^−^ ↔ MH_ads_ + OH (1)

MH_ads_ + H_2_O + e^−^ ↔ M + H_2_ + OH (2)

2MH_ads_ ↔ 2M + H_2_ (3)

where M indicates metal and Hads indicates adsorbed hydrogen. Reaction (1) (Volmer step) represents the electroreduction of water molecules by hydrogen adsorption onto the electrode; reaction (2) (Heyrovsky step) represents the electrochemical hydrogen desorption process, and reaction (3) (Tafel step) represents the production of H2 by chemical desorption of absorbed hydrogen atoms [35,36,37].

The LSV polarization curves of the pristine NF and NF* indicate overpotentials of 299 and 260 mV at 10 mA/cm^2^, respectively (Figure 5a and Table 2). The corresponding Tafel slopes are 147 and 161 mV/dec, as shown in Figure 5b. After depositing Ru, the overpotentials significantly decreased. The overpotential at 10 mA/cm^2^ further decreased from 36 to 25 mV with 95% Ar–5% O_2_ plasma treatment (sample Ru/NF*-AO). The Tafel slope of Ru/NF*-AO is 33 mV/dec. The HER kinetics follow the Volmer–Tafel mechanism [38,39].

To characterize the electrode/electrolyte interface and the corresponding processes, EIS measurements were performed at different selected overpotentials: 20, 50, 100, and 200 mV. At a cathodic overpotential of 20 mV, hydrogen evolution does not begin; at those of 50 mV and 100 mV, hydrogen production occurs at a very low rate; and at that of 200 mV, hydrogen is energetically generated [40]. Figure 5c presents the Nyquist plots of the Ru/NF*–AO catalyst at overpotentials of 200, 100, 50, and 20 mV versus RHE; the equivalent electrical circuit is shown in the inset. In the model circuit, R_s_ is the series resistance, R_ct_ is the charge-transfer resistance and CPE is the constant-phase element. The impedance properties are similar at different HER overpotentials, as shown in Figure 5c, suggesting the occurrence of similar electrochemical processes at all these overpotentials [41]. The results show that the electron transfer kinetics of the HER is faster with increasing overpotential, which is in accordance with the obtained polarization curves.

Figure 5d shows the Nyquist plots of pristine NF, NF*, Ru/NF*, Ru/NF*-A, Ru/NF*-AH, and Ru/NF*-AO catalysts in 1 M KOH at an overpotential of 200 mV vs. RHE; the inset shows magnified views of these plots. The R_ct_ values for Ru/NF*, Ru/NF*-A, Ru/NF*-AH, and Ru/NF*-AO are 0.4, 0.4, 0.6, and 0.2 Ω, respectively; these are much lower than those of pristine NF (17.1 Ω at an overpotential of 200 mV) and NF* (10.3 Ω at an overpotential of 200 mV). This implies a low charge-transfer resistance and highly efficient electron transport in all Ru-coated NF samples. Furthermore, the Ru/NF*-AO catalyst shows the lowest R_ct_ value, suggesting that the oxygen-containing plasma most significantly reduces the interfacial impedance.

Figure 5e shows the cyclic voltammetry (CV) potential curves of Ru/NF*-AO at scan rates of 20–300 mV/s. Figure 5f and Table 2 show the corresponding parameters for two times the double-layer capacitance (C_dl_) of the catalyst. The fitting linear slope is equivalent to that of two times the C_dl_ value [42,43]; it is frequently used to express the electrochemical surface area (ECSA) [44,45,46]. The C_dl_ value of NF* is increased nearly twofold compared to that of pristine NF. In addition, the C_dl_ of the catalyst was significantly improved after Ru deposition. A value of 95% Ar–5% O_2_ plasma treatment further increased the C_dl_ value and ECSA.

Previous studies have demonstrated that oxygen-containing plasma treatment could incorporate oxygen species into the surface with increased polarity [10,47]. This might explain why the electrocatalyst treated with oxygen-containing plasma has better performance in 1 M KOH aqueous solution. Furthermore, Ru catalysts with a small amount of lattice oxygen offer more suitable binding energies of oxygen intermediates for optimal activity [48]. These results reflect those of Yuanli et al. (2021) [49] who also found enriched defects in the surface-introduced lattice oxygen in the subsurface of Ru, leading to the overall optimization of the electronic structure and coordination environment of the active sites. The Ru surface model not only weakens the bonding strength of the catalyst and H but also accelerates water molecule dissociation. This can greatly contribute toward obtaining excellent HER activity. In accordance with the above results, the oxygen plasma treatment causing surface defects or a slight oxidation reaction is suggested to enhance the HER activity.

#### 3.5.2. PBS and H_2_SO_4_ Electrolyte

**Figure 6:**
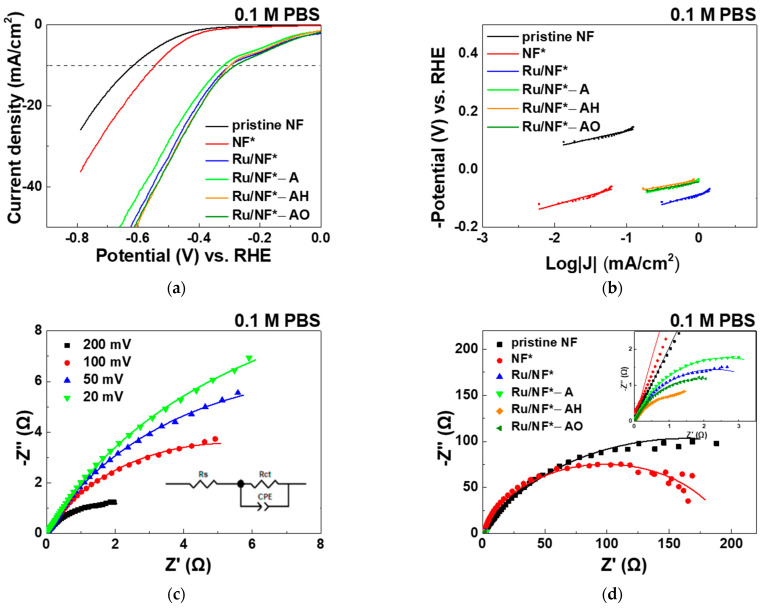
In 0.1 M PBS aqueous solution: (**a**) LSV polarization curves of pristine NF, NF*, Ru/NF*, Ru/NF*-A, Ru/NF*-AH, and Ru/NF*-AO toward HER. (**b**) Corresponding Tafel plots of HER. (**c**) Nyquist plots of the Ru/NF*-AO catalyst at four different applied overpotentials versus RHE. (**d**) Nyquist plots of pristine NF, NF*, Ru/NF*, Ru/NF*-A, Ru/NF*-AH, and Ru/NF*-AO catalysts at an overpotential of 200 mV versus RHE.

**Figure 7:**
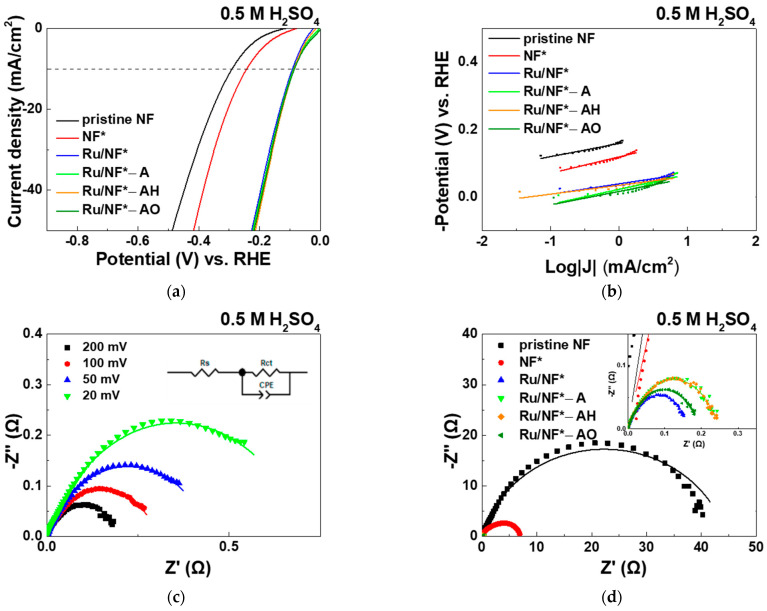
In 0.5 M H_2_SO_4_ aqueous solution: (**a**) LSV polarization curves of pristine NF, NF*, Ru/NF*, Ru/NF*-A, Ru/NF*-AH, and Ru/NF*-AO catalysts toward HER. (**b**) Corresponding Tafel plots of HER. (**c**) Nyquist plots of the Ru/NF*-AO catalyst at four different applied overpotentials versus RHE. (**d**) Nyquist plots of pristine NF, NF*, Ru/NF*, Ru/NF*-A, Ru/NF*-AH, and Ru/NF*-AO catalysts at an overpotential of 200 mV versus RHE.

Figure 6 and Table 3 show the electrochemical HER activity evaluated in 0.1 M PBS aqueous solution. Figure 7 and Table 4 shows the electrochemical HER activity in evaluated in 0.5 M H_2_SO_4_ aqueous solution. In 0.1 M PBS aqueous solution, the pristine NF, NF*, Ru/NF*, Ru/NF*-A, Ru/NF*-AH, and Ru/NF*-AO showed overpotentials (at 10 mA/cm^2^) of 617, 544, 300, 320, 300, and 281 mV, respectively (Figure 6a). In 0.5 M H_2_SO_4_ aqueous solution (Figure 7a), the pristine NF, NF*, Ru/NF*, Ru/NF*-A, Ru/NF*-AH, and Ru/NF*-AO show overpotentials (at 10 mA/cm^2^) of 291, 243, 94, 89, 87, and 86 mV, respectively. The overpotential decreased after H_2_SO_4_ cleaning. Similar to the case of 1 M KOH solution, Ru deposition also reduced the overpotential significantly in 0.1 M PBS and 0.5 M H_2_SO_4_ solutions. Oxygen-containing (95% Ar–5% O_2_) plasma treatment also produced electrodes with the smallest overpotential in these two cases.

In neutral aqueous solution, the water molecules are first dissociated to generate hydrogen atoms adsorbed onto the surface of the electrode, and then, the hydrogen atoms evolve to form H_2_ molecules [5,50]. Unlike in alkaline media, protons play an important role as a source for generating hydrogen molecules in acidic media, as indicated by the following three equations: Volmer: H^+^ + M + e^−^ → MH_ads_; Heyrovsky: MH_ads_ + H^+^ + e^−^ → M + H_2_ and Tafel: 2MH_ads_ → 2M + H_2_ [5,51]. The Tafel slope of pristine NF, NF*, Ru/NF*, Ru/NF*-A, Ru/NF*-AH and Ru/NF*-AO for neutral HER was in the interval of 45–69 mV/dec (Figure 6b), corresponding to a typical Volmer–Heyrovsky HER mechanism in which the electrochemical recombination represents the rate-determining step [5]. Moreover, the Tafel slope of the pristine Ru/NF*, Ru/NF*-A, Ru/NF*-AH, and Ru/NF*-AO in acidic media was approximately 30 mV/dec (Figure 7b), corresponding to a typical Volmer–Tafel HER mechanism.

To provide further insight into the relative enhancement of the HER catalytic activity for the Ru/NF*-AO catalyst, EIS analysis was performed at various HER overpotentials (20–200 mV) in 0.1 M PBS and 0.5 M H_2_SO_4_ aqueous solutions, and the Nyquist plots of the EIS responses are shown in Figure 6c and Figure 7c. As the overpotential increases, the R_ct_ decreases. The Nyquist plots of pristine NF, NF*, Ru/NF*, Ru/NF*-A, Ru/NF*-AH, and Ru/NF*-AO catalysts at an overpotential of 200 mV are shown in Figure 6d and Figure 7d. R_ct_ value was significantly decreased after cleaning with H_2_SO_4_ and depositing a Ru layer, in good agreement with the overpotential results. A lower R_ct_ value usually indicates faster electron transfer between electrocatalyst–electrolyte interface [41]. In 0.1 M PBS and 0.5 M H_2_SO_4_ aqueous solutions, Ru/NF* electrocatalysis changes slightly after three types of plasma treatments, as indicated by the R_ct_ values and overpotentials.

The long-term durability of Ru/NF*-AO in alkaline, neutral, and acidic solution was tested through LSV measurement, as shown in Figure S5. The overpotential at 10 mA/cm^2^ was stable during the measurement in alkaline and neutral solutions. The Ru/NF*-AO catalyst showed excellent durability and overpotential decay of approximately 29 and 22 mV after 12 h in 1 M KOH and 0.1 M PBS, respectively. Compared with the decay in 1 M KOH and 0.1 M PBS aqueous solutions, that in the 0.5 M H_2_SO_4_ aqueous solution was slightly higher, probably because of the corrosion of the electrode in acid.

## 4. Conclusions

Low-pressure 95% Ar–5% H_2_, pure Ar, and 95% Ar–5% O_2_ plasmas are used for post-treating Ru/NF. Ru/NF is then tested as a catalyst for HER. The overpotential at 10 mA/cm^2^ decreased from 36 to 25 mV with 95% Ar–5% O_2_ plasma treatment in alkaline solution. A low Tafel slope of 33 mV/dec was achieved under the Heyrovsky–Tafel mechanism of HER kinetics after oxygen-containing plasma treatment. The EIS results also indicate that 95% Ar–5% O_2_ plasma treatment significantly decreases the charge-transfer resistance, in agreement with the overpotential result. The Ru/NF*-AO catalyst showed excellent durability and overpotential decay of ~29 mV after 12 h in 1 M KOH.

## Figures and Tables

**Table 1:** Elemental analysis of EDS spectra of pristine NF, NF*, Ru/NF*, Ru/NF*-A, Ru/NF*-AH, and Ru/NF*-AO.

	Element (wt.%)				
	Ni	Ru	O	C	Cl
Pristine NF	96.1	-	3.9	-	-
NF*	91.8	-	8.2	-	-
Ru/NF*	37.0	49.4	5.6	7.0	1.0
Ru/NF*-A	49.0	42.7	2.8	4.9	0.7
Ru/NF*-AH	45.7	46.9	3.0	3.4	1.0
Ru/NF*-AO	34.3	56.5	5.3	3.0	1.1

After cleaning procedure with 0.1 M H_2_SO_4_, DI water, alcohol, and ACE. A: Plasma treatment with 100% Ar. AH: Plasma treatment with 95% Ar + 5% H_2_. AO: Plasma treatment with 95% Ar + 5% O_2_.

**Table 2:** Corresponding parameters of electrocatalyst in 1M KOH as calculated from Figure 5.

Electrocatalyst	Overpotential (mV) @Current Density 10 mA/cm^2^	Tafel Slope (mV/dec)	Rct (Ω)	2CdL (mF/cm^2^)
Pristine NF	299	147	17.1	1.12
NF*	260	161	10.3	2.19
Ru/NF*	36	44	0.4	44.60
Ru/NF*-A	50	68	0.4	27.85
Ru/NF*-AH	50	49	0.6	30.61
Ru/NF*-AO	25	33	0.2	51.41

After cleaning procedure with 0.1 M H_2_SO_4_, DI water, alcohol, and ACE. A: Plasma treatment with 100% Ar. AH: Plasma treatment with 95% Ar–5% H_2_. AO: Plasma treatment with 95% Ar–5% O_2_.

**Table 3:** Corresponding parameters of electrocatalysts in 0.1 M PBS as calculated from Figure 6.

Electrocatalyst	Overpotential (mV) @ Current Density 10 mA/cm^2^	Tafel Slope (mV/dec)	Rct (Ω)
pristine NF	617	56	327.2
NF*	544	68	197.2
Ru/NF*	300	69	4.6
Ru/NF*-A	320	55	5.3
Ru/NF*-AH	300	45	3.5
Ru/NF*-AO	281	45	3.9

After cleaning procedure with 0.1 M H_2_SO_4_, DI water, alcohol, and ACE. A: Plasma treatment with 100% Ar. AH: Plasma treatment with 95% Ar–5% H_2_. AO: Plasma treatment with 95% Ar–5% O_2_.

**Table 4:** The corresponding parameters of the electrocatalyst in 0.5 M H_2_SO_4_ aqueous solution calculated from Figure 7.

Electrocatalyst	Overpotential (mV) @ Current Density 10 mA/cm^2^	Tafel Slope (mV/dec)	Rct (Ω)
pristine NF	291	39	44.6
NF*	243	49	7.3
Ru/NF*	94	31	0.2
Ru/NF*-A	89	23	0.3
Ru/NF*-AH	87	35	0.3
Ru/NF*-AO	86	26	0.2

After cleaning procedure with 0.1 M H_2_SO_4_, DI water, alcohol, and ACE. A: Plasma treatment with 100% Ar. AH: Plasma treatment with 95% Ar–5% H_2_. AO: Plasma treatment with 95% Ar–5% O_2_.

## Data Availability

Not applicable.

## Supplementary Materials

The following supporting information can be downloaded at: https://www.mdpi.com/article/10.3390/ma15072603/s1, Figure S1: XPS spectra of C1s for the catalytic activity of (a) pristine NF, (b) NF*, (c) Ru/NF*, (d) Ru/NF*-A, (e) Ru/NF*-AH, (f) Ru/NF*-AO.; Figure S2: XPS spectra of Ru3d for the catalytic activity of (a) pristine NF, (b) NF*, (c) Ru/NF*, (d) Ru/NF*-A, (e) Ru/NF*-AH, (f) Ru/NF*-AO.; Figure S3: XPS spectra of O1s for the catalytic activity of (a) pristine NF, (b) NF*, (c) Ru/NF*, (d) Ru/NF*-A, (e) Ru/NF*-AH, (f) Ru/NF*-AO.; Figure S4: XPS spectra of Ni2p for the catalytic activity of (a) pristine NF, (b) NF*, (c) Ru/NF*, (d) Ru/NF*-A, (e) Ru/NF*-AH, (f) Ru/NF*-AO.; Figure S5: LSV polarization curves for HER catalyzed by Ru/NF*-AO before and after a durability test in (a) 1 M KOH, (a) 0.1 M PBS, and (c) 0.5 M H2SO4 aqueous solution.
