# Supplementary material for: Low-Pressure Plasma-Processed Ruthenium/Nickel Foam Electrocatalysts for Hydrogen Evolution Reaction
Source: Materials (Basel). 2022 Apr 1;15(7):2603. doi: 10.3390/ma15072603 (PMC9000553; doi:10.3390/ma15072603)
Supplement: Supplementary file 1 [file materials-15-02603-s001.zip › materials-1651279-supplementary.pdf]

(Supplementary Information)

Low-Pressure-Plasma-Processed Ruthenium/Nickel Foam Electrocatalysts for Hydrogen Evolution Reaction

Chen Liu[1,2], Chia-Yun Tseng[1,2], Ying-Chyi Wang[1,2], I-Chun Cheng[3,4], Jian-Zhang Chen[1,2,4,5,*]

[1] Graduate Institute of Applied Mechanics, National Taiwan University, Taipei City 10617, Taiwan
[2] Advanced Research Center for Green Materials Science and Technology, National Taiwan University, Taipei City 10617, Taiwan
[3] Graduate Institute of Photonics and Optoelectronics and Department of Electrical Engineering, National Taiwan University, Taipei City 10617, Taiwan
[4] Innovative Photonics Advanced Research Center (i-PARC), National Taiwan University, Taipei City 10617, Taiwan
[5] Graduate School of Advanced Technology, National Taiwan University, Taipei City 10617, Taiwan
***** Correspondence: jchen@ntu.edu.tw (J.Z.C.); Tel.: +886-2-33665694 (J.Z.C.)

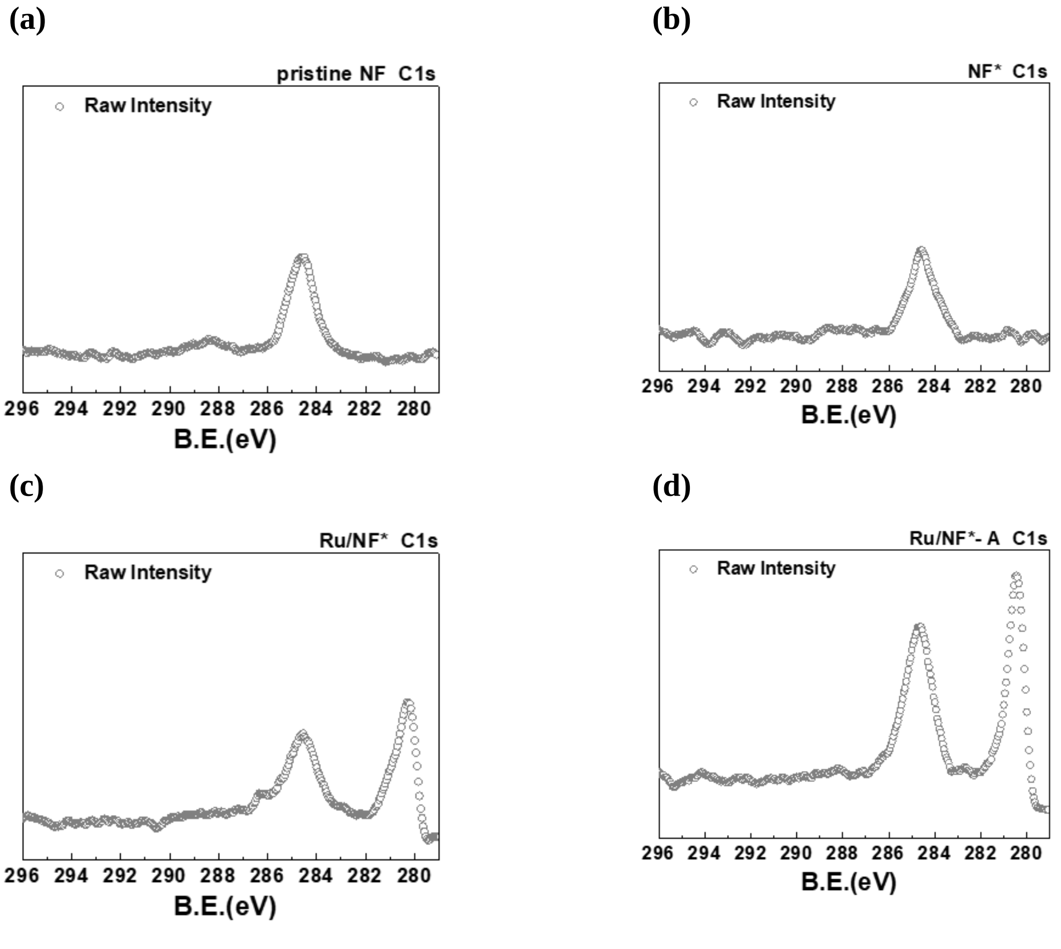

(e)

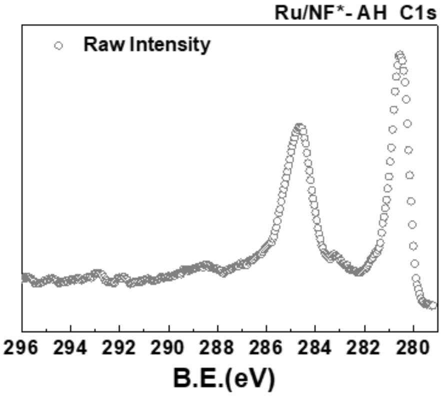

(f)

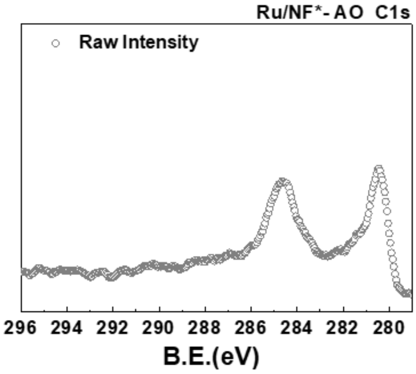

Figure S1. XPS spectra of C1s for the catalytic activity of (a) pristine NF, (b) NF*, (c) Ru/NF*, (d) Ru/NF*- A, (e) Ru/NF*- AH, (f) Ru/NF*- AO.

(a)

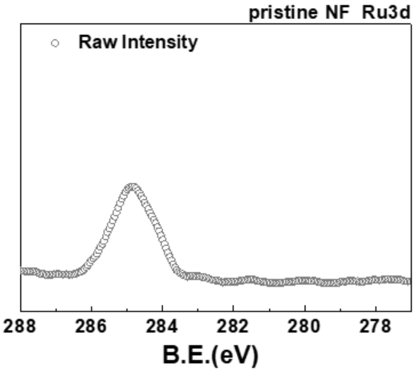

(b)

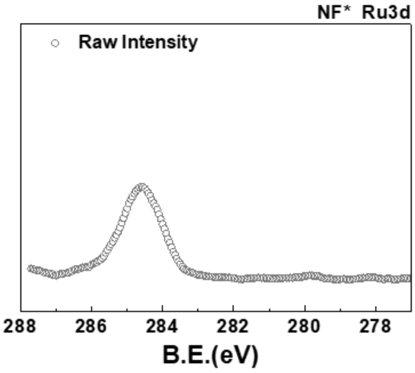

(c)

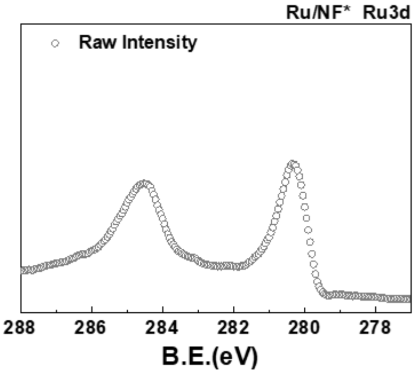

(d)

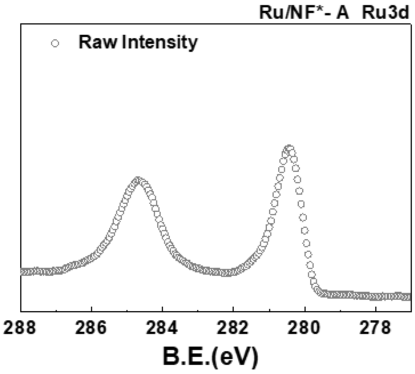

(e) (f)

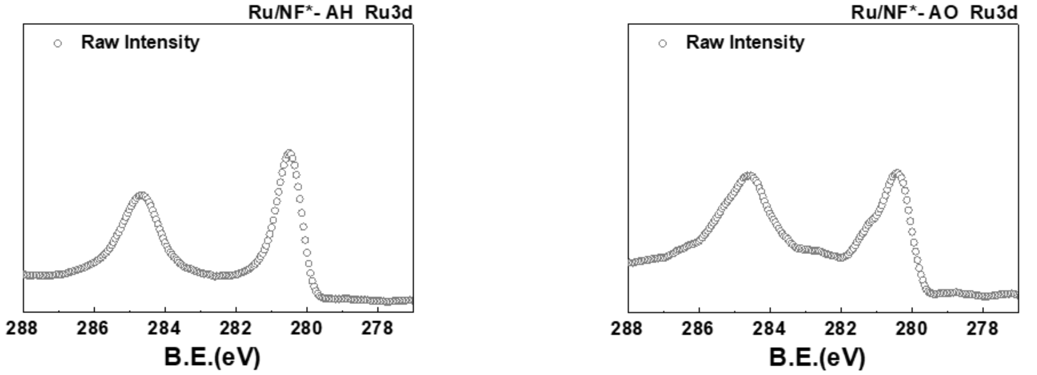

Figure S2 XPS spectra of Ru3d for catalytic activity of (a) pristine NF, (b) NF*, (c) Ru/NF*, (d) Ru/NF*- A, (e) Ru/NF*- AH, and (f) Ru/NF*- AO.

(a) (b)

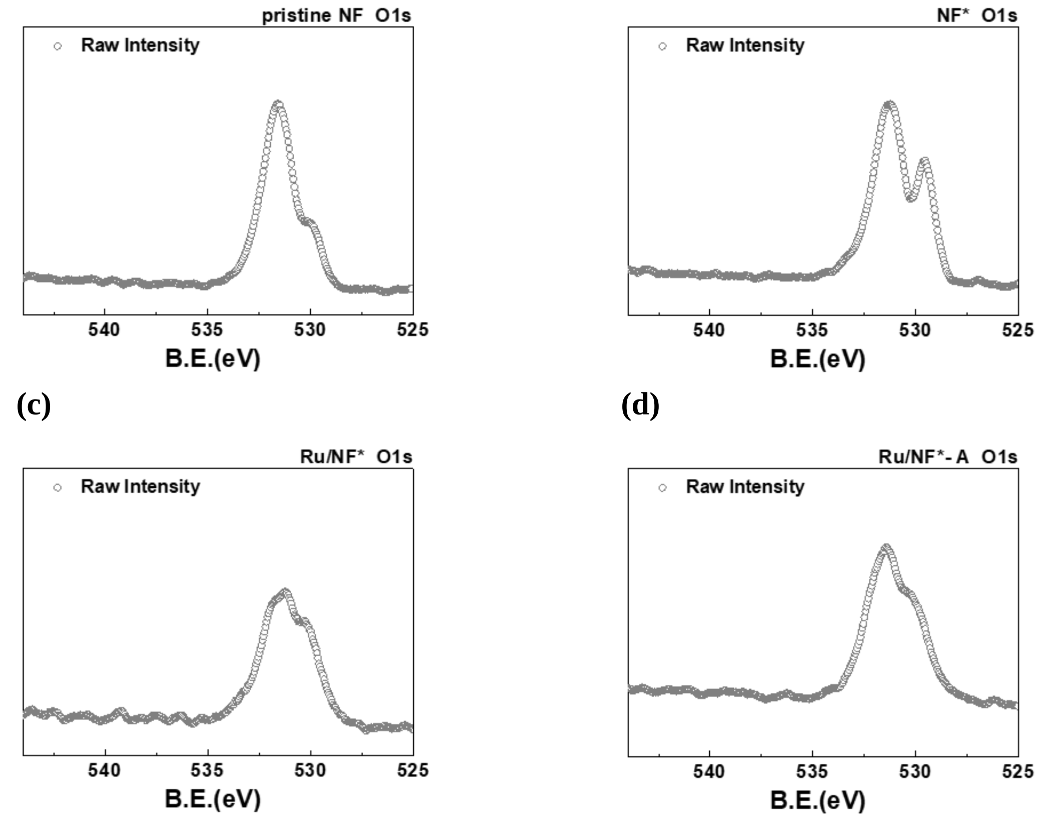

(e) (f)

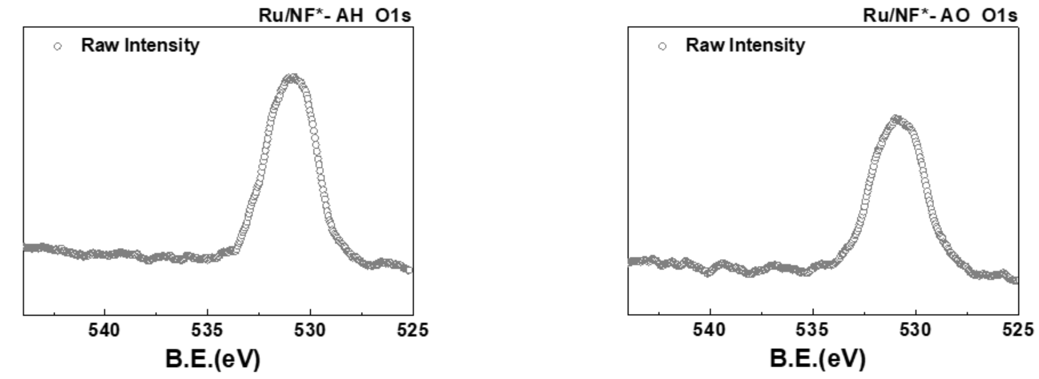

Figure S3 XPS spectra of O1s for catalytic activity of (a) pristine NF, (b) NF*, (c) Ru/NF*, (d) Ru/NF*-A, (e) Ru/NF*-AH, and (f) Ru/NF*-AO.

(a) (b)

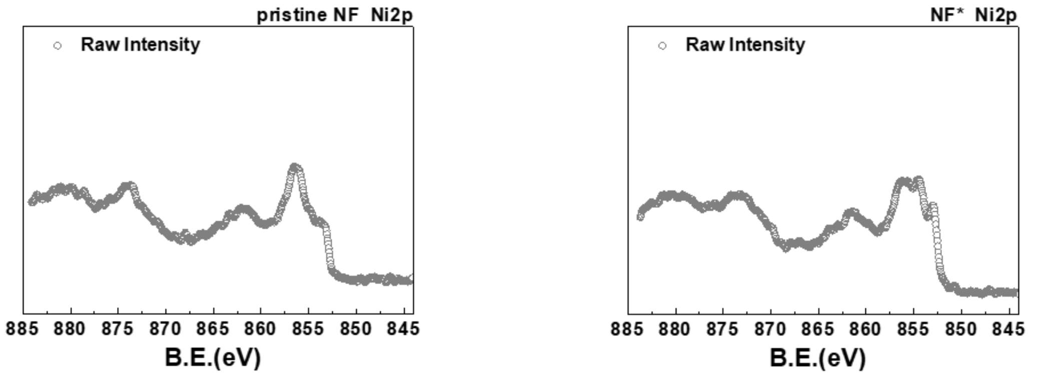

(c) (d)

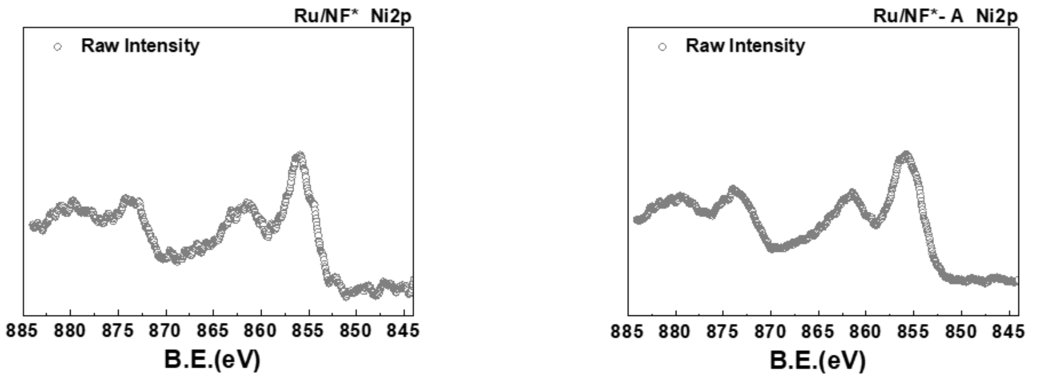

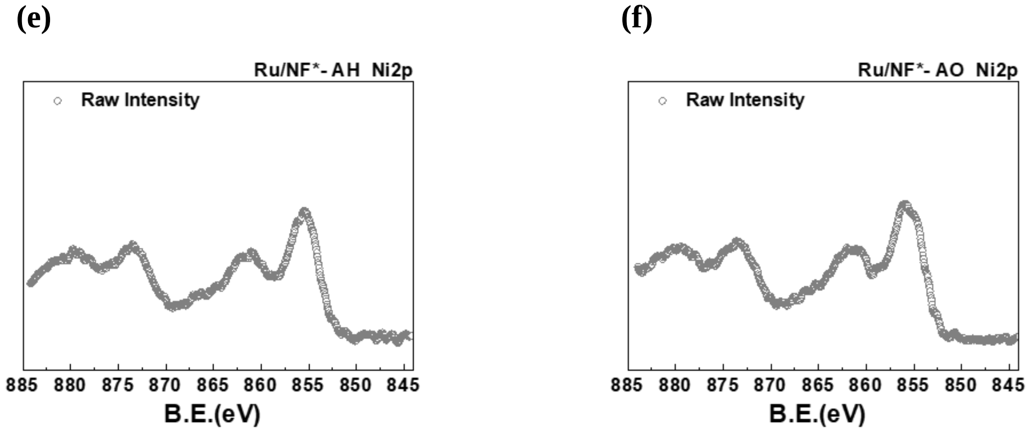

Figure S4 XPS spectra of Ni2p for catalytic activity of (a) pristine NF, (b) NF*, (c) Ru/NF*, (d) Ru/NF*-A, (e) Ru/NF*-AH, and (f) Ru/NF*-AO.

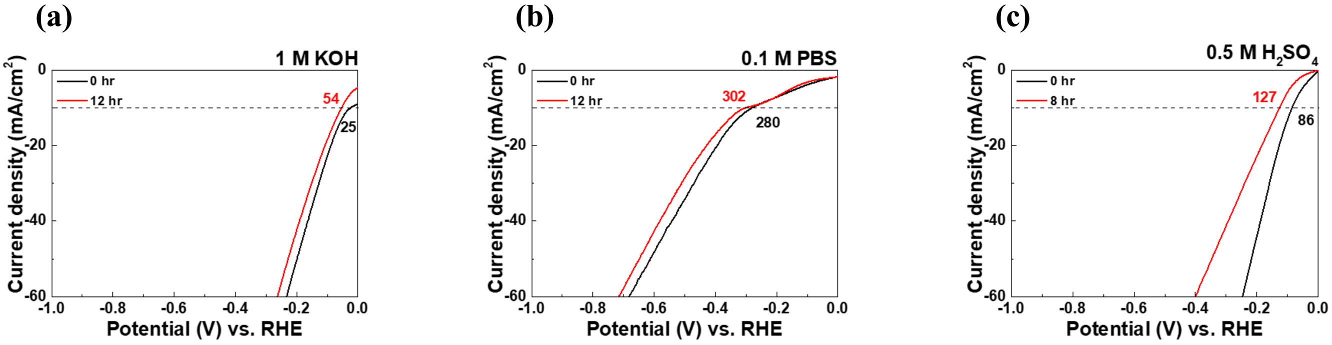

Figure S5 LSV polarization curves for HER catalyzed by Ru/NF*-AO before and after a durability test in (a) 1 M KOH, (a) 0.1 M PBS, and (c) 0.1 M H_2SO_4 aqueous solutions.
